# Unrecognized staphylococcal vertebral osteomyelitis leading to fatal outcome in a previously healthy patient

**DOI:** 10.1002/ccr3.1864

**Published:** 2018-11-20

**Authors:** Yazan Zayed, Bakr Swaid, Rahul Gupta, Tarek Haykal, Danielle Osterholzer

**Affiliations:** ^1^ Department of Internal Medicine, Hurley Medical Center Michigan State University Flint Michigan

**Keywords:** bacteremia, epidural abscess, infective endocarditis, vertebral osteomyelitis

## Abstract

New onset low back pain which is recalcitrant to usual treatment should be evaluated aggressively even in the absence of fever or neurologic deficits. Corticosteroids given for back pain will accelerate occult spinal infection and may mask symptomatology leading to more severe disease.

## 1 INTRODUCTION

Vertebral osteomyelitis is a serious condition which can progress to epidural abscess with subsequent high‐grade bacteremia and the development of infective endocarditis, central nervous system infection, or other disseminated foci. A high index of suspicion with appropriate treatment of the disease is essential to prevent fatal consequences.

Vertebral osteomyelitis can be acute, subacute, or chronic. The incidence of vertebral osteomyelitis (VO) increases with increasing age occurring more commonly after the age of 50 years. The most common presenting symptoms are back pain, fever, neurologic impairment, and constitutional symptoms. Identifying VO at an early stage is necessary to initiate proper treatment and to prevent systemic complications. This is a case of VO which was diagnosed late in the disease course with a tragic outcome.

## 2 CASE PRESENTATION

**Figure 1:**
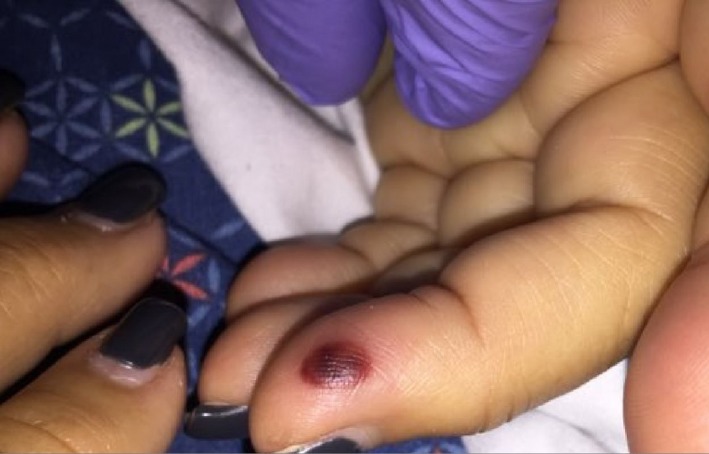
Janway lesion of the middle finger

A 71‐year‐old Caucasian female with no known past medical history complained of insidious onset low back pain that became constant and severe, associated with generalized weakness and diffuse pain over the course of few days. The patient sought medical care at other facilities on two occasions and each time was prescribed symptomatic treatment including narcotic pain medications and a course of oral steroids. She had no fever or chills. Her family noted that she was increasingly confused therefore; they brought her to this hospital after 2 weeks of complaints. On examination, she was afebrile with tachycardia. She was lethargic but arousable. No meningismus was present. A pan‐systolic murmur was present as well as a red painless raised lesion on the pad of her left fourth finger (Figure 1). She had midline lower back tenderness with preserved power and sensation in her lower limbs.

**Figure 2:**
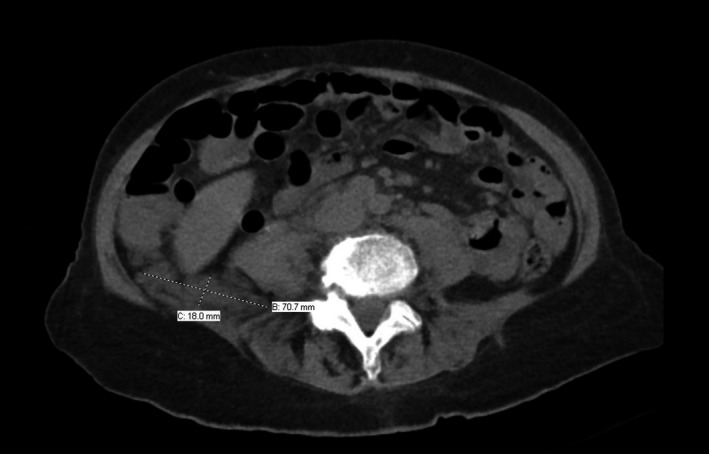
CT scan showing fluid collection and psoas abscess

Laboratory investigation showed leukocytosis with a left shift, elevated C‐reactive protein, and erythrocyte sedimentation rate as well as acute kidney injury. A lumbar puncture revealed 100 white blood cells (100% monocytes) with elevated protein and low glucose. Both her blood and spinal fluid grew methicillin‐sensitive *Staphylococcus aureus* in <12 hours. She was treated with nafcillin. Computed tomography (CT) of the spine revealed a fluid collection in the retroperitoneum concerning for psoas abscess (Figure 2). Echocardiography revealed a mitral valve vegetation with severe regurgitation. Numerous foci compatible with acute embolic infarcts were evident on brain magnetic resonance imaging (MRI) while lumbar spine MRI showed vertebral osteomyelitis and discitis with an epidural abscess displacing the spinal cord.

The patient's condition deteriorated rapidly as she developed severe septic shock with multi‐organ failure. She was not a candidate for spinal or cardiac surgery given her severity of illness. Unfortunately, she died just shy of a month after her initial complaints began.

## 3 DISCUSSION

Low back pain is the most common symptom of VO. Fever is present only in 35%‐60% of cases; therefore, fever absence does not exclude the disease.^1^ *Staphylococcus aureus* is the most common causative agent and accounts for 32%‐67% of cases. Hematogenous seeding is the most common mode of acquisition and the source of primary bacteremia is often subclinical and is only detected in about 50% of the cases. Interestingly, infective endocarditis is diagnosed in up to a third of cases with VO.^2^ Less commonly, patients who are known to have endocarditis are found secondarily to have VO. Most patients with hematogenous pyogenic VO have underlying medical conditions or intravenous drug abuse. Psoas abscess is a common consequence of direct extension of infection from the lumbar spine and when found should prompt spinal imaging if no other etiology is clear.^3^

This case highlights the sometimes‐occult nature of VO. Hematogenous seeding of the spine with staphylococci can occur even through small violations in skin integrity and the initial portal of entry may not be evident. Patients may complain only of worsening back pain without fever or other signs of infection. Prednisone given for a presumed diagnosis of sciatica or muscle inflammation can lead to acceleration of occult infection. In this case, spinal osteomyelitis progressed quickly to epidural and psoas abscesses, meningitis, endocarditis, embolic stroke, and death.

## 4 CONCLUSIONS

Vertebral osteomyelitis requires a high index of suspicion to diagnose. Corticosteroids should be used with extreme caution in those with back pain. Failure to recognize this entity early on may have dire consequences including death.

## CONFLICT OF INTEREST

None declared.

## AUTHOR CONTRIBUTION

BS and RG: performed the literature search. YZ and TH: drafted the manuscript. YZ and DO: contributed to the final approval of the manuscript.
